# Understanding Pain in Polycystic Ovary Syndrome: Health Risks and Treatment Effectiveness

**DOI:** 10.1101/2024.10.15.24315513

**Published:** 2024-10-16

**Authors:** Tess Cherlin, Stephanie Mohammed, Sasha Ottey, Katherine Sherif, Shefali S. Verma

**Affiliations:** 1 Department of Pathology and Laboratory Medicine, Perelman School of Medicine, Philadelphia, PA, United States.; 2 PCOS Challenge: The National Polycystic Ovary Syndrome, Atlanta, GA, USA.; 3 Department of Medicine, Sidney Kimmel Medicine College, Thomas Jefferson University, Philadelphia, PA, USA

**Keywords:** PCOS, polycystic ovary syndrome, pain, abdominal pain, pelvic pain, dysmenorrhea, health disparities, future health outcomes

## Abstract

Polycystic ovary syndrome (PCOS) is a prevalent endocrine disorder in women, often accompanied by various symptoms including significant pain, such as dysmenorrhea, abdominal, and pelvic pain, which remains underexplored. This retrospective study examines electronic health records (EHR) data to assess the prevalence of pain in women with PCOS. Conducted on May 29, 2024, using data from 120 Health Care Organizations within the TriNetX Global Network, the study involved 76,859,666 women from diverse racial backgrounds. The analysis focused on the prevalence of pain among women with PCOS, both overall and in those prescribed PCOS-related medications. Relative risk ratios (RR) were calculated for future health outcomes and stratified by self-reported race. The study found that 19.21% of women with PCOS experienced pain, with the highest prevalence among Black or African American (32.11%) and White (30.75%) populations. Both the PCOS and PCOS and Pain cohorts exhibited increased RR for various health conditions, with significant differences noted across racial groups for infertility, ovarian cysts, obesity, and respiratory diseases. Additionally, women with PCOS who were treated with PCOS-related medications showed a decrease in pain diagnoses following treatment. In conclusion, this study highlights the critical need to address pain in the diagnosis and management of PCOS due to its significant impact on patient health outcomes.

## Introduction

According to the World Health Organization (WHO), polycystic ovary syndrome (PCOS) affects approximately 8–13% of women of reproductive age, with an alarming 70% of affected individuals remaining undiagnosed globally(Organization, 28 June 2023). The assessment of PCOS has been substantiated by multiple guidelines (Azziz et al., 2016; Teede et al., 2010)and has undergone refinement since its initial description by Stein and Leventhal in 1935(Stein & Leventhal, 1935). Standard diagnostic criteria have evolved through international efforts, including conferences convened by the National Institutes of Health (NIH) in 1990(Zawadri, 1992), the ESHRE/ASRM-sponsored PCOS consensus workshop group in Rotterdam in 2003 and 2004(ESHRE & Group, 2004), and the International Evidence-based Guideline for the Assessment and Management of Polycystic Ovary Syndrome in 2018, most recently updated in 2023(Mousa & Tay, 2023; Teede et al., 2018).

Recommendations for assessing PCOS encompass a multifaceted approach, including the evaluation of irregular menstrual cycles, ovulatory dysfunction, biochemical and clinical hyperandrogenism, ultrasound findings, serum Anti-Mullerian Hormone (AMH) levels, and various other factors such as ethnic disparities, cardiovascular disease risk, menopausal status, impaired glucose tolerance, and risk of type 2 diabetes mellitus (T2DM)(Mousa & Tay, 2023; Teede et al., 2018). Additionally, screening and managing psychological manifestations, implementing lifestyle interventions, and adhering to pharmacological treatment principles are integral aspects of PCOS management(Mousa & Tay, 2023; Teede et al., 2018). While the diagnostic criteria for PCOS primarily focus on reproductive and metabolic manifestations, the substantial burden of *pain* experienced among women with PCOS is a critical factor that warrants effective prevention and management of the disease.

The assessment of pain in PCOS necessitates a multidimensional approach, incorporating self-reported scales, clinical evaluation, and possibly imaging techniques to elucidate the underlying etiology and severity. Several commonly utilized assessment tools incorporate evaluations of pain among women with PCOS. The Polycystic Ovary Syndrome Health-Related Quality of Life Questionnaire (PCOSQ), developed by Cronin et al. in 1998, assesses various domains, including painful menstrual cycles(Cronin et al., 1998). Additionally, the SF-36 scale, examines eight dimensions of health, including bodily pain(McHorney et al., 1993). Women with PCOS across diverse demographic backgrounds have consistently reported lower SF-36 scores specifically in the domain of bodily pain(Drosdzol et al., 2007; Elsenbruch et al., 2003; Hahn et al., 2005; Li et al., 2011). Furthermore, the Menorrhagia Outcomes Questionnaire, developed by Lamping et al. in 1998, evaluates both heavy menstrual bleeding (HMB) and the associated pain(Lamping et al., 1998). Despite the validation of these instruments, they may not comprehensively capture key symptoms expressed by patients with PCOS, especially those related to dysmenorrhea, abdominal, or pelvic pain. Insufficient data exist to highlight the prevalence of pain reported by women both before and after a PCOS diagnosis, as well as any associations between this pain and the condition itself and its long-term effects. To address this gap in research, we propose an investigation utilizing health records to shed light on this underexplored aspect of PCOS.

Electronic health records (EHRs) have become indispensable for managing vast amounts of clinical data, including patient demographics, medical history, medications, allergies, laboratory test results, vital signs, and imaging reports, as well as genetic information obtained from patient genomes when available. Given that EHRs contain comprehensive information about patient care, including the progression of signs and symptoms, severity, comorbidities, and treatments, they provide invaluable resources for conducting large-scale retrospective studies. EHR-based studies have been particularly valuable in assessing the prevalence of conditions that are often underdiagnosed or misdiagnosed in women(Kruse et al., 2018; Maletzky et al., 2022; Penrod et al., 2023). The temporal aspect of clinical events, such as the onset of symptoms, treatment administration, and follow-up visits, can also be mined from EHRs, providing crucial insights into disease trajectories and treatment efficacy(Zhao et al., 2017).

Pain, particularly in the context of PCOS, remains an underexplored area of research. By leveraging EHR data we can identify women with PCOS who have reported dysmenorrhea, abdominal, and pelvic pain. The objective of this research is to use EHR and look at longitudinal data retrospectively to determine the pain reported by women with PCOS and to determine associations with other factors. This study aims to explore the prevalence of pain in women with and without PCOS, examine the relative risk of long-term health outcomes in those with and without pain, and assess the impact of medications on pain symptoms. By analyzing longitudinal EHR data, we seek to uncover patterns associated with comorbidities, infertility, and medication usage, and explore their potential effects on pain thresholds. Our approach will provide insights into the relationship between pain symptoms and PCOS contributing to a better understanding of the condition and potentially improving patient care.

## Methods

### Study Design

The data used in this study was collected on May 29^th^, 2024 at 3:00pm EST from the TriNetX^20^ Global Network, which provided access to electronic medical records (diagnoses, procedures, medications, laboratory values, genomic information) from approximately 140,962,260 million patients from 120 healthcare organizations.

### Cohort Definitions

A retrospective cohort analysis was conducted for patients with PCOS and patients with PCOS and Pain. Patients who were identified as cases (met inclusion and exclusion criteria) were compared to their respective controls. Description of inclusion and exclusion criteria for each cohort can be found in **Supplemental Table 1 – Figure supplement 1**.

For the PCOS cohort, PCOS was defined as either having a PCOS diagnosis (ICD 10 E28.2), or an irregular menstruation (ICD 10 N92.6) and hirsutism (ICD 10 L68.0) diagnosis, or an irregular menstruation (ICD 10 N92.6) and androgen excess (ICD 10 E28.1) diagnosis. PCOS controls were defined as having a physical examination (ICD 10 Z00.0) and none of the PCOS case criteria. PCOS participants also had to satisfy stringent exclusion criteria to avoid confounders. Exclusion criteria for PCOS consisted of Maternal care for benign tumor of corpus uteri (ICD 10 O34.1), Leiomyoma of uterus (ICD 10 D25), Endometriosis (ICD 10 N80), Polyp of corpus uteri (ICD 10 N84.0), and Female pelvic inflammatory disease unspecified (ICD 10 N73.9). Description of inclusion and exclusion criteria for each cohort can be found in **Table 1 - Figure supplement 1**.

For the PCOS and Pain cohort, PCOS was defined the same as above. PCOS and Pain cases were defined as patients with a PCOS case as well as being diagnosed for either abdominal and pelvic pain (ICD 10 R10) or dysmenorrhea (ICD 10 N94.6) +/− three months from their first PCOS diagnosis. PCOS and Pain controls were defined as patients with PCOS but no pain diagnoses. Description of inclusion and exclusion criteria for each cohort can be found in **Table 1- Figure supplement 1**.

To compare cohorts (cases / controls), the first documented encounter or PCOS (case / controls) or PCOS and Pain (case / controls) was defined as an “index event” in TriNetX. Index events are the specific dates a patient satisfies all selected cohort criteria. Baseline characteristics are all assessed *before* the index event while all health outcomes are assessed *after* the index event. **Figure 1- Figure supplement 2** shows a graphical representation of this relationship among index events and outcomes specified in this study.

### Propensity Score Matching

**Figure 1.**
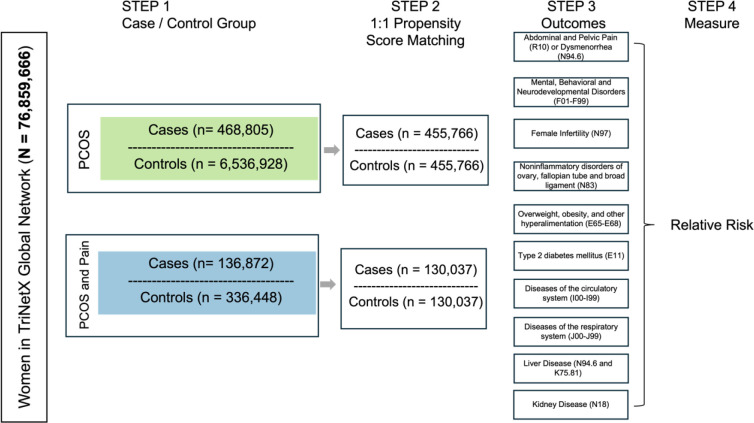
Analysis pipeline. Analysis pipeline to calculate relative risk ratios (RR) for future health outcomes in PCOS (green) and PCOS and Pain (blue) cohorts for the 76859,666 women queried. STEP 1 shows the number of women in the case and controls for both the PCOS (green) and PCOS and Pain (blue) cohorts. STEP 2 shows the number of cases and controls after 1:1 propensity score matching. STEP 3 shows the different future health conditions that were considered for future health outcomes. STEP 4 shows that the final step is calculating the relative risk for the future health outcomes.

The TriNetX platform uses a cohort matching method called 1:1 propensity score matching(Austin, 2011). For each cohort analysis, cases were matched on the following criteria: age at the index event, self-reported race, overweight, obesity, and other hyperalimentation (ICD 10 E65-E69) status, type 2 diabetes mellitus (T2D) (ICD 10 E11) status, essential (primary) hypertension (ICD 10 I10) status, and hyperlipidemia, unspecified (ICD 10 E78.5) status. Baseline conditions were assessed up to one day before the index event. Figure 1 shows the number of patients in each case and control cohort both at baseline and after propensity score matching.

### Future Health Outcomes

For each of the five cohorts listed above, we calculated relative risk ratios (RR) with 95% confidence intervals for 10 future health outcomes: mental, behavioral and neurodevelopmental disorders (ICD 10 F01-F99), female infertility (ICD 10 N97), noninflammatory disorders of ovary, fallopian tube and broad ligament (ICD 10 N83), Obesity (ICD 10 E65-E68), Type 2 Diabetes (ICD 10 E11), diseases of the circulatory system (ICD 10 I00-I99), diseases of the respiratory system (J00-J99), abdominal and pelvic pain (ICD 10 R10) or dysmenorrhea, unspecified (ICD 10 N94.6), nonalcoholic steatohepatitis (ICD 10 K75.81) or fatty liver, not elsewhere classified (ICD 10 K76.0), and chronic kidney disease (ICD 10 N18). The RR for future health outcomes was calculated on participants who satisfied the 1:1 propensity score matching criteria (above). Differences in relative risks were calculated for significance by calculating the difference of two estimates(Altman & Bland, 2003). Future health outcomes were only considered if their first occurrence was at least 3-months after the index event. Figure 1 shows the health outcomes assessed after 1:1 propensity score matching(Austin, 2011; Guo & Fraser, 2014; Haukoos & Lewis, 2015). These analyses were done within the TriNetX platform, and no individual-level data was extracted from the platform.

### Self-reported Race Stratified Sub-Analysis

We did a follow-up analysis looking at health outcomes for patients with PCOS and PCOS and Pain compared to matched controls stratified by self-reported race and ethnicity. The following race categories are present in the TriNetX platform: American Indian or Alaskan Native, Asian, Black or African American, Native Hawaiian or Other Pacific Islander, Other, White, and Unknown Race. For our analysis, we included the following four race categories: Asian, Black or African American, and Other (American Indian or Alaskan Native or Native Hawaiian or Other Pacific Islander, or Other), and White **(Figure 2 - Figure supplement 3).** Due to the small population sizes of American Indian or Alaskan Native or Native Hawaiian or Other Pacific Islander, or Other, we decided to combine these four self-reported race groups together into one “Other” population. For the PCOS cohorts we looked at the pain health outcome stratified by self-reported race (**Figure 2A - Figure supplement 3**). For the PCOS and Pain cohorts, we looked at ovarian cysts, infertility, obesity, T2D, mental health conditions, circulatory diseases, respiratory diseases, liver disease, and kidney disease stratified by self-reported race (**Figure 2B - Figure supplement 3**).

### PCOS Medication Sub-analysis

We performed a follow-up analysis looking at the number of patients with PCOS who were documented as having pain before being prescribed three common PCOS medications (systemic contraceptives (VA: HS200), metformin (RxNorm 6809), or spironolactone (RxNorm 9997) as shown in **Figure 3 - Figure supplement 4**. Three cohorts were created for patients were included if they had a PCOS diagnosis (described above) *and* 1) ever had a systemic contraceptives prescription but not a metformin or spironolactone prescription, 2) ever had a metformin prescription but not a systemic contraceptives or spironolactone prescription, and 3) ever had a spironolactone but not a systemic contraceptives or metformin prescription. For patients with PCOS, we counted the number of participants who had a diagnosis code for either dysmenorrhea or abdominal and pelvic pain before the index event. The index event was defined as a participant having a PCOS diagnosis and a medication prescription at the same time. We then counted the number of patients with PCOS who reported either dysmenorrhea or abdominal and pelvic pain after the index event. We further compared the change in prevalence before and after the indexed event.

## Results

### Demographics of women with PCOS in the TriNetX Global Network

We first identified participants with PCOS and associated comorbidities. The demographics and characteristics of both the PCOS and non-PCOS cohorts are detailed in **Table 2 - Figure supplement 5**. We queried the 76,859,666 women of any age from 120 Health Care Organizations (HCOs) in the TriNetX Global Network for PCOS and associated comorbidities. After applying stringent inclusion/exclusion criteria for PCOS and subsequent controls (see Methods), we were left with a total population of 7,005,733. We found a 7% (n = 468,805 prevalence of PCOS at an average age of 28.7 (SD ± 9.49) in this population. Of those participants with PCOS, 4.61%, 11.35%, 22.73%, and 61.32% self-identified as Asian, Black or African American, Other, or White respectively (**Table 2 - Figure supplement 5**).

We then examined the prevalence of PCOS-associated comorbidities represented in the PCOS cohort. We observed that 16.11% of patient with PCOS had a diagnosis code for obesity, 6.23% had a diagnosis code for essential hypertension, 4.01% had a diagnosis code for T2D, and 3.24% had a diagnosis code for hyperlipidemia (**Table 2 - Figure supplement 5**).

We also investigated the prevalence of other women’s health conditions related to PCOS such as infertility and ovarian cysts. **Table 2 - Figure supplement 5** shows that women with PCOS had a 2.53% prevalence of infertility, a 3.55% prevalence of ovarian cysts.

The TriNetX platform also allow to easily survey the diagnoses that are most prevalent among a cohort population. As can be observed in **Table 2 - Figure supplement 5**, that respiratory system diseases, mental health conditions, and digestive system diseases were most prevalent among the PCOS cohort (24.24%, 20.97%, and 19.40% respectively).

Since many women with PCOS are prescribed medications to help manage symptoms associated with the disease, we aimed to gain a deeper understanding of the prevalence of PCOS-prescribed medications in the PCOS cohort. We found that 12.76% of the PCOS cohort were prescribed systemic oral contraceptives, 7.07% were prescribed metformin, and 2.77% were prescribed spironolactone (**Table 2 - Figure supplement 5**).

A focus of our paper is understanding the impact pain has on women with PCOS, therefore, we looked at the prevalence of both dysmenorrhea and abdominal and pelvic pain. We observed that overall, there was a 19.21% prevalence of pain (2.46% prevalence of dysmenorrhea, 16.75% prevalence of abdominal or pelvic pain) (**Table 2 - Figure supplement 5**).

### Demographics of women with PCOS and Pain in the TriNetX Global Network

**Figure 2.**
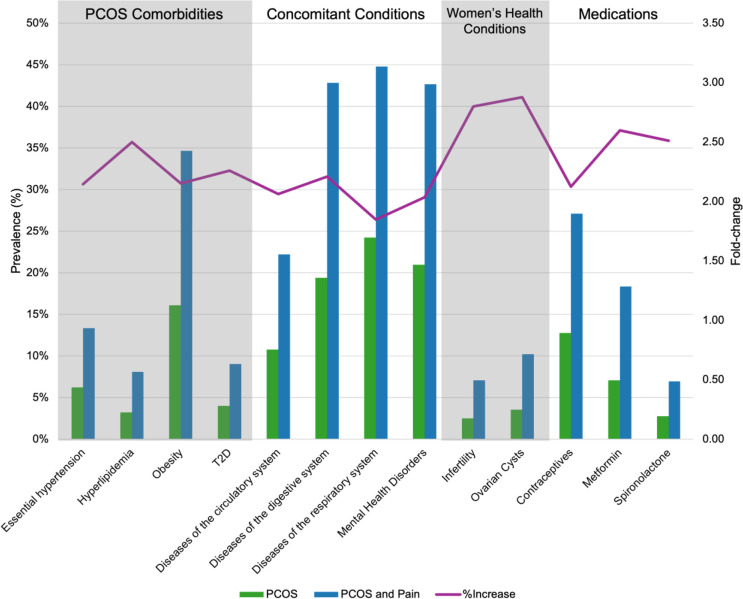
Prevalence of conditions and medications associated with PCOS and Pain. Barplots show the prevalence (%) (left y-axis) of different diseases associated with PCOS (green) and PCOS and Pain (blue) (x-axis). Purple line indicates the prevalence fold-change between the PCOS and PCOS and Pain cohorts (right y-axis).

As noted above, 19.21% of women with PCOS also had a pain diagnosis, encompassing either dysmenorrhea or pelvic and abdominal pain. We first examined the demographics of the study participants using data from the TriNetX Global Network. **Table 3 - Figure supplement 6** shows the comprehensive demographic results, highlighting the distribution of pain diagnoses for age, race, and other relevant demographic factors. This table provides a clear view of the demographic characteristics and their potential influence on the prevalence of pain among individuals with PCOS. Similar to the PCOS cohort, participants with PCOS and Pain had an average age of 29.02 (SD ± 9.23) and among those 3.57% women were Asian, 12.54% Black or African America, 19.24% categorized as Other, and 64.65% self-reported as White (**Table 3 - Figure supplement 6**). However, when we looked at the prevalence of PCOS and Pain compared to controls (PCOS without pain) within a self-reported race group, we observed that the highest prevalence of PCOS and Pain was 32.11% in the Black or African American population followed by 30.75% in the White population, 24.75% in the Other population, and 22.74% in the Asian population (**Table 4 -- Figure supplement 7**). With respect to PCOS comorbidities, the cohort of individuals with both PCOS and Pain exhibited a higher prevalence of comorbid conditions compared to the entire population of individuals with PCOS. Specifically, 34.68% of PCOS and Pain participants had an obesity diagnosis, 13.36% had an essential hypertension diagnosis, 9.05% had a T2D diagnosis, and 8.09% had a hyperlipidemia diagnosis. These high prevalences represent a respective increase of 18.57%, 7.13%, 5.04%, and 4.85% compared to all participants with PCOS. Notably, as illustrated in Figure 2, there is at least a two-fold increase in the prevalence of each comorbid condition among those with both PCOS and Pain. This substantial increase highlights the heightened risk and burden of comorbidities within the PCOS and Pain cohort.

We observed a similar trend with respect to the most highly co-occurring diseases with PCOS. Diseases of the respiratory system, digestive system, and mental health conditions had prevalences of 44.81%, 42.85%, and 42.68%, respectively, which is at least a 2-fold increase for all three conditions compared to the entire PCOS cohort (Figure 2). We also see a 2-fold increase in the prevalence of infertility (7.08%) and ovarian cysts (10.22%) in the PCOS and Pain cohort when we compared to the PCOS cohort as shown in Figure 2. Further, there is at least a 2-fold increase in prescriptions for all three common PCOS symptom-management medications in PCOS and Pain cohort compared to PCOS cohort (Figure 2).

### Relative Risk of Future Health Outcomes for all PCOS vs. PCOS and Pain

**Figure 3.**
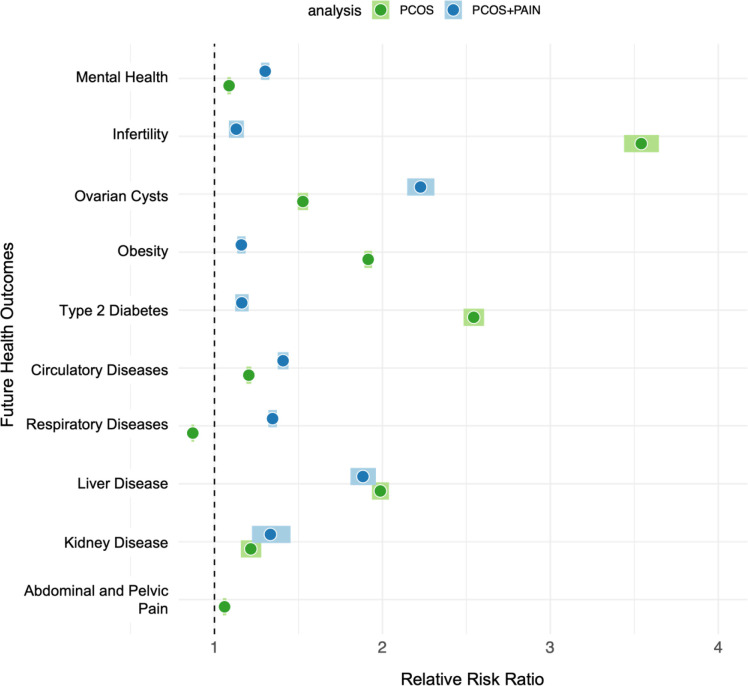
Relative risk ratios for future health outcomes associated with PCOS and Pain. Relative risk ratios (RR) (x-axis) for future health outcomes (y-axis) for both PCOS (green) and PCOS and Pain (blue) cohorts. Darker hued circles indicate RR, while lighter hued boxes indicate the 95% confidence intervals. The black dashed line is set 1 and is the threshold for RR, where > 1 is increased RR and < 1 is decreased RR.

Given that PCOS symptoms first manifest in puberty and during reproductive years, we aimed to assess the relative risk for patients with PCOS, and those with both PCOS and Pain, developing future health outcomes. We calculated the RR for matched PCOS patient cohorts with their respective controls and PCOS and Pain patient cohorts with their respective controls (see Methods). Results are visualized in Figure 3 and cohorts counts, RRs, and p-values for differences in risk ratios are provided in **Table 5 - Figure supplement 8**.

In Figure 3 we see the RR for both participants within the PCOS cohort (green) for 10 health outcomes and PCOS and Pain cohort (blue) for 9 health outcomes. Aside from respiratory disease, the PCOS cohort has significantly increased risk for developing the following future health outcomes compared to matched controls: mental and behavioral health, infertility, ovarian cysts, obesity, T2D, circulatory diseases, liver disease, and pain. The PCOS and Pain cohort meanwhile has significantly increased risk for developing all of the following future health outcomes compared to their matched controls: mental and behavioral health, infertility, ovarian cysts, obesity, T2D, circulatory diseases, liver disease, and kidney disease.

While almost all of the RR are increased for case cohorts compared to match controls, a few results stand out as being particularly interesting. Infertility, for example, a common complication associated with PCOS, has a RR of 3.49 (95% CI 3.40–3.60) in PCOS cases overall. While still statistically significant, the RR is much lower, 1.12 (95% CI 1.08–1.17), in PCOS and Pain cases. This difference in relative risks has a p-value of 6.0×10^−484^ (**Table 5 - Figure supplement 8**). On the other hand, ovarian cysts have a RR of 1.52 (95% CI 1.49–1.55) in PCOS cases overall but an even higher RR in PCOS and Pain cases (RR=2.31, 95% CI 2.23–2.39). This relative risk difference is also significant with a p-value of 1.25×10^−45^ (**Table 5 -- Figure supplement 8**). In addition, mental health conditions, circulatory diseases, and respiratory diseases all had higher RRs in PCOS and Pain cases vs. controls compared to PCOS cohort cases compared to controls (Figure 3, **Table 5 - Figure supplement 8**). We see that mental health conditions, circulatory diseases, and respiratory diseases are 1.32 (95% CI 1.29–1.34), 1.41 (95% CI 1.38–1.44), and 1.35 (95% CI 1.33–1.38) in the PCOS and Pain cohort compared to 1.08 (95% CI 1.07–1.09), 1.21 (95% CI 1.19–1.22), and 0.87 (95% CI 0.86–0.88) in the entire PCOS cohort at statistical significance (Figure 3, **Table 5 - Figure supplement 8)**.

PCOS and PCOS and Pain cohorts were at comparable increased risk for developing future liver disease and kidney disease. For liver disease, the RR in the PCOS cohort was 2.03 (95% CI 1.98–2.08) while the RR in the PCOS and Pain cohort was 1.89 (95% CI 1.82–1.97). For kidney disease, the RR of the PCOS cohort was 1.22 (95% CI 1.16–1.28) while the RR for the PCOS and Pain cohort was 1.35 (95% CI 1.24–1.46) (Figure 3). The relative risk difference was statistically significant for liver disease (p-value = 3.91×10^−06^) but not for kidney disease (p-value = 4.66×10^−02^) **(Table 5 - Figure supplement 8)**.

Obesity and T2D had increased risk for the PCOS cohort compared to the PCOS and Pain cohort. Obesity had a RR of 1.94 (95% CI 1.92–97) in the PCOS cohort compared to 1.15 (95% CI 1.12–1.17) in the PCOS and Pain cohort. Meanwhile, the RR for T2D was 2.59 (95% CI 2.53–2.66) in the PCOS cohort compared to 1.16 (95% CI 1.12–1.20) in the PCOS and Pain cohort (Figure 3). The relative risk difference was statistically significant for both obesity (p-value = 7.78×10^−297^) and T2D liver disease (p-value = 6.72×10^−307^) **(Table 5 - Figure supplement 8)**.

### Relative Risk of Future Health Outcomes Stratified by Self-Reported Race

**Figure 4:**
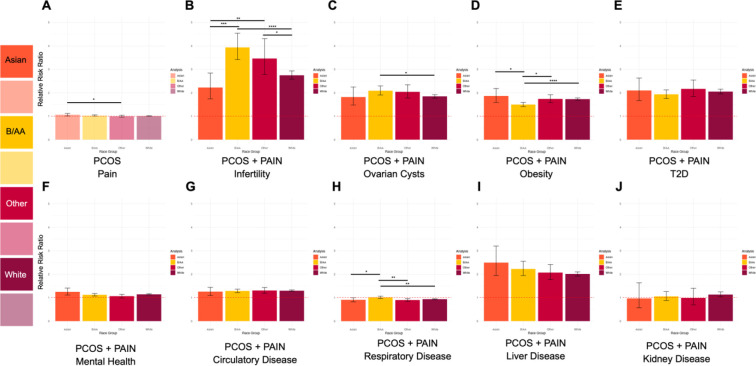
Parts A-J. Self-reported race-stratified relative risk ratios for future health outcomes. Relative risk ratios (RR) for future health outcomes (y-axis) stratified by self-reported race. Colors represent different self-reported race groups (x-axis): Asian (orange), Black or African American (yellow), Other (red), White (purple). **A.** Light hues indicate RR calculation for entire PCOS cohort. **B-J.** Dark hues indicate RR calculation for PCOS and Pain cohorts. Error bars represent the 95% confidence intervals. Significant differences between RR are represented by asterisks (*), where p-value ≤ 0.05 = *, p-value ≤ 0.005 = **, p-value ≤ 0.0005 = ***, and p-value ≤ 0.00005 = ****. Red dashed lines is set 1 and is the threshold for RR, where > 1 is increased RR and < 1 is decreased RR.

Since we observed an increased risk for future health outcomes for both the PCOS and PCOS and Pain cohorts compared to their respective controls, we next investigated if there were any race-specific risks (self-reported from EHR) for these outcomes. We stratified the PCOS case and control cohorts by self-reported race and calculated RR for pain (dysmenorrhea or abdominal and pelvic pain). There was no significant difference in RR between self-reported race groups for PCOS cases compared to matched controls (Figure 4A).

When examining the PCOS and Pain cohort, we observe race-specific differences in RR for a number of future health outcomes. Figure 4B shows that infertility has the highest RR in the Black or African American cohort with PCOS and Pain (RR = 3.94, 95% CI 3.41–5.54) followed by Other (RR = 3.46, 95% CI 2.78–4.31), White (RR = 2.75, 95% CI 2.58–2.93), and Asian (RR = 2.22, 95% CI 1.74–2.84). The RR difference between the self-reported Asian and Black cohorts, Asian and Other cohorts, and Black or African American and White cohorts, and Other and White cohorts were all statistically significant with a p-value less than 0.05 (Figure 4B, **Table 6 - Figure supplement 9, Table 7 - Figure supplement 10**). On the other hand, liver diseases (Figure 4I) had the highest RR in the Asian cohort (RR = 2.50, 95% CI 1.95–3.20) with PCOS and Pain followed by Black or African American (RR = 2.22, 95% CI 1.94–2.55), Other (RR = 2.07, 95% CI 1.77–2.41), and White (RR = 2.01, 95% CI 1.92–2.10), however, none of these RR differences were statistically significant across self-reported race groups (**Table 6 - Figure supplement 9, Table 7 - Figure supplement 10**). Ovarian cysts (Figure 4C) had the highest RR in the Black or African American cohort (RR = 2.09, 95% CI 1.91–2.30) and Other cohort (RR = 2.04, 95% CI 1.78–2.34) compared to the Asian (RR = 1.82, 95% CI 1.48–2.46) and White (RR = 1.84, 95% CI 1.77–1.92) cohorts. Meanwhile, there were similar trends for obesity (Figure 4D) and T2D (Figure 4E) where the Black or African American cohort had lower RRs than the other three self-reported race cohorts (**Table 6 - Figure supplement 9**). There were significant RR difference between the Asian and Black or African American cohorts (p=1.12×10^−02^), Black or African American and Other cohorts (p=9.38×10^−03^), and Black or African American and White cohorts (p=2.17×10^−05^) for obesity but not T2D (Figure 4D, **Table 7 - Figure supplement 10**). Mental health disorders (Figure 4F) had an increased RR for the Asian cohort (RR=1.25, 9%% CI 1.11–1.41) while the other self-reported race groups did not have significantly increased RRs. However, there was a significant difference in RR for the Asian and Other cohorts (p-value = 2.02×10^−02^) Oppositely, kidney disease (Figure 4D) had a slightly increased RR (RR=1.13, 9%% CI 1.03–1.24) in the White cohort while the other self-reported race groups did not have significantly increased RRs (**Table 6 - Figure supplement 9**). Circulatory disease (Figure 4G) had increased RRs in all four self-reported race groups, but there was no significant difference among them (**Table 7 - Figure supplement 10**). And finally, respiratory disease (Figure 4H) had decreased RRs in Asian (RR=0.91, 9%% CI 0.83–0.99), Other (RR=0.90%, CI 0.85–0.96), and White (RR=0.93, 9% CI 0.92–0.95) cohorts but no increased or decreased risk in the Black or African American cohort (RR=1.01, 9%% CI 0.91–1.07) (**Supplemental Table 6**). The difference in RR between the Black or African American cohort and the three other self-reported race groups were statistically significant with a p-value less than 0.05 (Figure 4H, **Table 7 - Figure supplement 10**).

### Medications prescribed to patients with PCOS may modify pain prevalence

**Figure 5.**
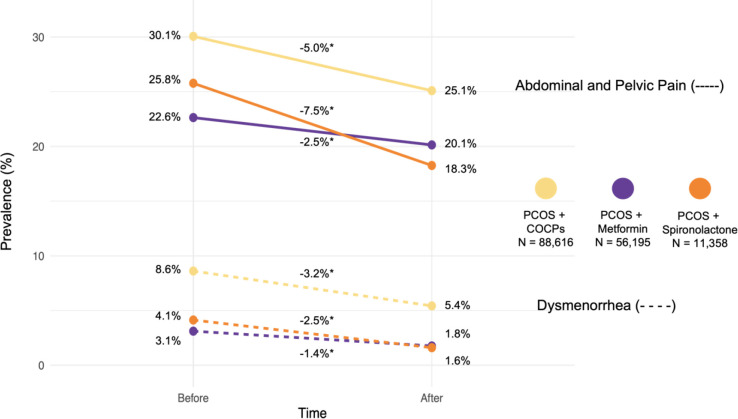
Prevalence of Pain for women with PCOS before and after medications. Prevalence (%) changes (y-axis) of pain for women with PCOS cohort before and after prescription of COCPs (yellow), metformin (purple), and spironolactone (orange) (x-axis). Analysis was done separately for abdominal and pelvic pain (solid lines) and dysmenorrhea (dashed lines).

Since women with PCOS are often prescribed medications to help their PCOS symptoms, we aimed to investigate if there were any changes in the pain diagnoses after being prescribed systemic contraceptives (COCPs), metformin, or spironolactone. For patients with PCOS who were prescribed each of the three medications exclusively, we calculated the percent who reported dysmenorrhea or abdominal and pelvic pain both before and after the prescription (see Methods). We found that there were 88,616 women with PCOS who were prescribed systemic contraceptives, 56,195 prescribed metformin, and 11,358 prescribed spironolactone. The prevalence of abdominal and pelvic pain diagnosis was 6–8X greater than that of a dysmenorrhea diagnosis for PCOS participants before they were prescribed PCOS-related medications (Figure 5).

As can be observed in Figure 5, participants with PCOS reported abdominal and pelvic pain at a prevalence of 30.1%, 25.8%, and 22.6% respectively before their first prescription of COCPs, metformin, and spironolactone. At least 3-months after being prescribed a PCOS-associated medication, we observe a significant reduction in the prevalence of abdominal and pelvic pain. After PCOS participants were prescribed all three medications we observed a significant reduction in the prevalence of pain for all three medications. Spironolactone has the largest reduction of pain prevalence with a −7.5% reduction of pain diagnosis after prescription compared to before, followed by COCPs (−5%) and metformin (−2.5%). Similar results are observed with dysmenorrhea. While lower overall, there was also a deceased prevalence of pain for all three medications, the prevalence for dysmenorrhea was 8.6%, 4.1% and 3.1% for participants with PCOS prescribed COCPs, spironolactone and metformin respectively. Unlike with abdominal and pelvic pain, COCP prescriptions were associated with the largest decrease in dysmenorrhea prevalence (−3.2%), followed by spironolactone (−2.5%), and (−1.4%).

## Discussion

Polycystic ovary syndrome is the most prevalent endocrine disorder among women(Mousa & Tay, 2023; Walters et al., 2018). Diagnosis and treatment plans are customized based on the symptoms presented by women. However, an important yet often overlooked variable is pain, which may manifest as dysmenorrhea, abdominal, or pelvic pain. The use of EHR data has facilitated access to patient records containing longitudinal clinical information, utilizing the readily available International Classification of Diseases (ICD) codes^16,(Wu et al., 2016)^. Our study aimed to elucidate the prevalence and impact of pain among individuals with PCOS, as well as to investigate the relative risk of future health outcomes and the effectiveness of commonly prescribed medications on pain. Firstly, we observed a significantly higher prevalence of pain among women with PCOS compared to those without the condition. Specifically, 19.21% of women with PCOS reported experiencing pain, compared to 15.8% in the non-PCOS cohort. This increased prevalence reveals the substantial burden of pain as a symptom of PCOS, which often goes underreported and undertreated. Our demographic analysis of women with PCOS and Pain also revealed a difference in diagnosis of pain across self-reported race groups and was especially high in the Black or African American population (32.11%) and White population (30.75%). These findings suggest that pain is a significant, yet often under-recognized, symptom of PCOS that can vary across different demographic groups. The high prevalence of pain underscores the need for healthcare clinicians to routinely assess and address pain in the management of PCOS, particularly in racially diverse populations. The diversity in pain perception and reporting among different racial groups can be influenced by a variety of factors, including genetic differences, cultural attitude towards pain, access to healthcare, and socioeconomic status. Women of different racial groups often experience different severities in pain(Portenoy et al., 2004). This can lead to disparities in pain management and treatment outcomes(Campbell & Edwards, 2012; Jamieson & Steege, 1996). Additionally, cultural differences may also affect how individuals report pain and their willingness to seek medical help(Hadjiconstantinou et al., 2017).

PCOS manifests with many other concomitant conditions.(Anagnostis et al., 2018; Asuncion et al., 2000; Balen et al., 2016; Escobar-Morreale et al., 2011; Hadjiconstantinou et al., 2017; Kitzinger & Willmott, 2002; Patel, 2018). Our study revealed that women with PCOS and Pain have at least a 2-fold increased prevalence of other health conditions at baseline compared to women with PCOS in general. The prevalence of obesity in the PCOS and Pain cohort was 34.68% compared to a 16.11% prevalence in the entire PCOS cohort Excess abdominal visceral fat is well-documented to increase inflammation(Després, 2012) and PCOS is considered a pro-inflammatory condition linked with cardiovascular disease (CVD) and T2D. This inflammation underlies obesity, CVD, insulin resistance (IR) (Abraham Gnanadass et al., 2021; Osborn & Olefsky, 2012). Our data show that 22.23% of patients with PCOS and Pain also had a diagnosis for disease of the circulatory system and 9.05% of PCOS and Pain patients had a T2D diagnosis. These results underscore the health challenges faced by individuals dealing with both PCOS and Pain issues necessitating treatment approaches that address both the syndrome itself and its accompanying symptoms.

Women with PCOS are significantly at risk for future health outcomes such as infertility, T2D, coronary heart disease, dyslipidemia, depression, non-alcoholic fatty liver disease, and obstructive sleep(Anagnostis et al., 2018; Ávila et al., 2014; Chaudhuri, 2023; McGowan, 2011; Patel, 2018; Zore et al., 2017). Our results also highlight specific risks for different subgroups (PCOS overall and PCOS and Pain). In the overall PCOS cohort, the highest risks are for infertility (RR = 3.49) and T2D (RR = 2.59). However, in patients with PCOS and Pain, the highest risks are for ovarian cysts (RR = 2.31) and liver disease (RR = 1.89). Ovarian cysts are a hallmark feature of polycystic ovarian morphology (PCOM) which is caused by immature/ arrested follicles that do not ovulate and cause a “string of pearls” appearance and enlarging of the ovaries(Adashi et al., 2023; Tsilchorozidou et al., 2004). Ovarian cysts have long been disputed by the PCOS research community as not being associated with PCOS and therefore, not being associated with pain. However, the magnitude of this risk as shown in our results underscores the importance of regular monitoring and appropriate management strategies for patients presenting with both PCOS and pain symptoms. PCOS is known to be linked with non-alcoholic fatty liver disease (NAFLD)(Butt & Devi, 2024; Kumarendran et al., 2018; Torres & Harrison, 2016). This association between PCOS and pain and liver disease may be explained by the shared metabolic disturbances common to both PCOS and NAFLD, such as insulin resistance and dyslipidemia(Georgescu, 2022; Qu et al., 2013; Torres & Harrison, 2016). The presence of chronic pain could potentially exacerbate these metabolic imbalances through various mechanisms, including altered stress responses and lifestyle factors(Kivimäki et al., 2023). These findings suggest that patients with PCOS who also experience chronic pain may represent a distinct phenotype with unique risk profiles. Additionally, the increased risk for future health conditions in the PCOS and Pain cohort also suggest that pain may be an important marker for identifying individuals at future health outcomes, necessitating more vigilant monitoring and proactive intervention.

Lastly, our analysis also delved into the impact of common PCOS medications on pain management. We found that the prescribing of COCPs, metformin, and spironolactone was associated with a reduction in reported pain symptoms. Specifically, individuals who received these medications showed a 3.68% average decreased prevalence of pain diagnoses after treatment, suggesting that these medications may also manage pain symptoms in individuals with PCOS. A recent publication looked at the association of PCOS-related medications with adverse drug reactions (ADRs) for women with PCOS and found that metformin and COCPs was significantly associated with abdominal pain (Sidra et al., 2019). However, this study did not measure the association with ADRs with pain before and after the prescription of PCOS medication. These results offer insights for application showing that efficient pharmacological management of PCOS symptoms can also help alleviate associated pain. Additionally, the efficacy of these medications in reducing pain, specifically spironolactone and COCPs, which are prescribed in PCOS for their antiandrogenic effects, may suggest hyperandrogenism to be a contributor to increased pain in PCOS, and a potential target for addressing pain in PCOS. Furthermore, the advantages of these treatments may be beneficial, not just in managing typical PCOS symptoms, but also in tackling the significant burden of pain experienced by many women with PCOS highlights a valuable role in drug repurposing.

Our study has limitations that need to be considered when interpreting the findings. Firstly, relying on ICD codes to identify pain and other health outcomes may not capture the range of experiences and clinical intricacies. While these codes offer an approach to data collection, they might not fully reflect variations in pain severity or the personal experiences of those, with PCOS(Kataria & Ravindran, 2020). Although extensive, the use of EHR data may still contain gaps or discrepancies that could impact the accuracy of our results(Madden et al., 2016). Furthermore, since this study is observational, by nature it cannot establish a causal relationship between PCOS, pain and future health outcomes. Moreover, the demographic variations observed—especially the higher occurrence of pain among individuals—could be influenced by socio-economic factors, access to healthcare, nutrition and other unmeasured variables. In addition, self-reported race was not captured the same globally as it is not a variable that is coded by HCOs world-wide. Lastly, TriNetX captures only medication prescriptions, which does not allow our analysis to consider adherence issues, dosage differences, or concurrent treatments that may influence the outcomes observed. Future research should focus on overcoming these limitations through studies with detailed clinical assessments and a broader range of demographic and socio-economic factors.

## Conclusion

Various pain subtypes can profoundly affect the daily lives of PCOS patients. Due to limited research in clinical and laboratory settings, the effects and underlying mechanisms of pain remain unclear. Our study highlights the significant prevalence and impact of pain in women with PCOS, revealing critical differences across racial groups and underscoring the heightened risk for future health complications in those experiencing pain. These findings emphasize the importance of comprehensive pain assessment, management and inclusion as guidelines in the standard care of PCOS, with a particular focus on addressing racial disparities. Additionally, the observed effectiveness of medications such as systemic oral contraceptives, metformin, and spironolactone in reducing pain symptoms provides valuable insights for clinical practice, suggesting that these treatments can offer dual benefits in managing both PCOS and associated pain. Dysmenorrhea, abdominal, and pelvic pain are common experiences in women with PCOS, in the absence of pelvic-related conditions that can contribute to this type of pain such as pelvic inflammatory disease, endometriosis, and fibroids. It is crucial to distinguish between pain originating from PCOS and Pain arising from comorbidities to ensure appropriate management and targeted treatment strategies for improving the quality of life in affected individuals.

## Data Availability

All summary-level data has all been included in this manuscript.

## Impact Statement

Insufficient data exist on the prevalence of pain in women with a PCOS diagnosis, and its associations with future health outcomes. Among, 444,348 women with PCOS in the TriNextX Global Network, 19.21% have dysmenorrhea, abdominal, and pelvic pain. Women with PCOS and Pain are at increased risk for developing ovarian cysts, infertility, T2D, and fatty liver disease and are at further risk when stratified by self-reported race groups.
